# Supplementary material for: Dogs and humans share biomarkers of mortality
Source: bioRxiv. 2025 Aug 25:2025.08.20.671317. Preprint. [Version 1] doi: 10.1101/2025.08.20.671317 (PMC12407799; doi:10.1101/2025.08.20.671317)
Supplement: Supplement 2 — Supplementary Table 1. Cohort Summary [file media-2.pdf]

PMID	study	cohort	sex (%female)	age	N subjects	deaths	freq. death	follow up (yrs)	follow up measure
this study	this study	Precision Cohort of the Dog Aging Project	48.6	mean=5.3 (SD=3.3)	937	104	0.11	2.64	average
31431621	Deelen et al 2019	Pravastatin in elderly individuals at risk of vascular disease	0.515	75.3	5329	467	0.09	2.76	average
31431621	Deelen et al 2019	Leiden Longevity Study	0.613	97.35	843	823	0.98	4.03	average
31431621	Deelen et al 2019	United Kingdom Adult Twin Registry	unknown	64.58	1996	58	0.03	4.32	average
31431621	Deelen et al 2019	Avon Longitudinal Study of Parents and Children	unknown	47	4351	17	0.00	5.69	average
32751974	Ottosson et al 2020	Malmö Preventive Project	23.6	70.5	369	118	0.32	7.7	average
31431621	Deelen et al 2019	Dietary, Lifestyle, and Genetic determinants of Obesity and Metabolic syndrome	0.532	52.39	4816	190	0.04	7.73	average
31431621	Deelen et al 2019	Alpha Omega Cohort	0.246	69.21	568	157	0.28	7.79	average
31431621	Deelen et al 2019	Estonian Biobank	0.626	46.1	10,988	912	0.08	7.97	average
31431621	Deelen et al 2019	Cooperative Health Research in the Region of Augsburg	0.513	60.89	1790	123	0.07	8.02	average
31431621	Deelen et al 2019	The Rotterdam Study	0.581	75	2963	1254	0.42	8.28	average
31651959	Balasubramanian et al 2020	Women's Health Initiative Hormone Therapy	100	range 50 to 180	1355	685	0.51	9.1	median
31651959	Balasubramanian et al 2020	Women's Health Initiative Observational Study	100	range 50 to 180	943	417	0.44	10.6	median
31431621	Deelen et al 2019	Erasmus Rucphen Family Study	0.549	50.44	680	107	0.16	10.67	average
31431621	Deelen et al 2019	Leiden Longevity Study	0.554	70.93	2241	191	0.09	11.76	average
40107652	Fernández-Duval et al 2025	Mediterranean diet for primary prevention of cardiovascular diseases	57.5	range 55 to 80	1878	457	0.24	12.2	median
39504246	Sebastiani_2024	Long Life Family Study	54	24 to 110, median 74	1267	unknown	NA	15	minimum
31431621	Deelen et al 2019	National FINRISK Study	0.503	48.29	7603	1213	0.16	16.7	average
25864806	Cheng et al 2015	Framingham Offspring Study	unknown	unknown	2,327	439	0.19	17.4	total
32751974	Ottosson et al 2020	Malmö Diet and Cancer-Cardiovascular Cohort	44.9	59.5	374	180	0.48	18.3	average
26956554	Yu et al 2016	Atherosclerosis Risk in Communities	57.7	~52-56 (range of means)	1887	671	0.36	22.5	average
37717037	Wang et al 2023	Health Professional Follow-Up Study	0	unknown	1,620	4288*	NA	22.60	median
37717037	Wang et al 2023	Nurses' Health Study I	100	unknown	6,883	4288*	NA	22.60	median
37717037	Wang et al 2023	Nurses' Health Study II	100	unknown	3,131	4288*	NA	22.60	median
29390044	Huang et al 2018	Alpha-Tocopherol, Beta-Carotene Cancer Prevention	0	range 50 to 69	620	435	0.70	28	total
